# School-Based Programs to Prevent and Reduce Alcohol Use among Youth

**Published:** 2011

**Authors:** Melissa H. Stigler, Emily Neusel, Cheryl L. Perry

**Keywords:** Alcohol and other drug use (AODU), alcohol consumption, alcohol abuse, age of AODU onset, underage drinking, adolescent, risk factors, individual risk factors, social environmental risk factors, elementary school, middle school, high school, school-based prevention, school-based intervention

## Abstract

Schools are an important setting for interventions aimed at preventing alcohol use and abuse among adolescents. A range of school-based interventions have been developed to prevent or delay the onset of alcohol use, most of which are targeted to middle-school students. Most of these interventions seek to reduce risk factors for alcohol use at the individual level, whereas other interventions also address social and/or environmental risk factors. Not all interventions that have been developed and implemented have been found to be effective. In-depth analyses have indicated that to be most effective, interventions should be theory driven, address social norms around alcohol use, build personal and social skills helping students resist pressure to use alcohol, involve interactive teaching approaches, use peer leaders, integrate other segments of the population into the program, be delivered over several sessions and years, provide training and support to facilitators, and be culturally and developmentally appropriate. Additional research is needed to develop interventions for elementary-school and high-school students and for special populations.

Because alcohol use typically begins during adolescence (Office of the Surgeon General 2006) and because no other community institution has as much continuous and intensive contact with underage youth, schools can be an important setting for intervention. This article describes school-based approaches to alcohol prevention, highlighting evidence-based examples of this method of intervention, and suggests directions for future research. This summary primarily is based on several recent reviews focusing on alcohol prevention among underage youth conducted by Foxcroft and colleagues (2002), Komro and Toomey (2002), and—the most comprehensive and critical review of this field to date—Spoth and colleagues (2008, 2009). Although these previous reviews addressed interventions in a variety of contexts (e.g., families, schools, and communities), the present article highlights key findings specific to school-based interventions.

## Characteristics of School-Based Alcohol Prevention Programs

Rates of initiation of drinking rise rapidly starting at age 10 (i.e., grades 4 and 5) and peak between ages 13 and 14 (i.e., grades 8 and 9). At that point, more than 50 percent of adolescents report ever having consumed alcohol in their lifetime (Kosterman et al. 2000). Given this natural history of alcohol use in adolescence, most school-based programs have been developed for and delivered in middle schools; programs aimed at elementary schools (especially grades 3 to 5) and high schools are less common (Spoth et al. 2008, 2009). Of particular concern to contemporary research with underage youth is heavy drinking, including harmful behaviors, such as binge drinking and drunkenness.

The primary goal of school-based alcohol prevention programs is to prevent or delay the onset of alcohol use, although some programs also seek to reduce the overall prevalence of alcohol use. Interventions earlier in life (i.e., during elementary school) target risk factors for later alcohol use (e.g., early aggression) because alcohol use itself is not yet relevant to this age group (Spoth et al. 2008, 2009). Any reduction in alcohol-related behavior is assumed to lead to subsequent reductions in alcohol-related problems (e.g., injuries or alcohol dependence), although the latter often are not measured in primary prevention studies (Foxcroft et al. 2002).

School-based alcohol interventions are designed to reduce risk factors for early alcohol use primarily at the individual level (e.g., by enhancing student’s knowledge and skills), although the most successful school-based programs address social and environmental risk factors (e.g., alcohol-related norms) as well. Some school-based programs focus on the general population of adolescents (i.e., are universal programs), whereas others target adolescents who are particularly at risk (i.e., are selective or indicated programs). The research literature on the efficacy of school-based alcohol prevention programs is large, encompassing several decades of study (Foxcroft et al. 2002; Komro and Toomey 2002; Spoth et al. 2008, 2009). The most recent review by Spoth and colleagues (2008, 2009) provides several examples of effective school-based programs, which will be discussed in detail below. Not all school-based alcohol prevention programs for youth are effective, however. The review by Foxcroft and colleagues (2002), especially, emphasizes this point with regard to long-term (3 years or more) outcomes of primary prevention efforts such as school-based programs.

## Examples of Evidence-Based, School-Based Alcohol Prevention Programs

The review by Spoth and colleagues (2008, 2009) provides support for the efficacy of school-based programs, at least in the short term (defined as at least 6 months after the intervention was implemented). This review considered alcohol prevention interventions across three developmental periods (i.e., younger than age 10 years, age 10 to 15 years, and age 16 years or older), aligned with reviews of other etiologic work during the same developmental stages (Masten et al. 2009; Zucker et al. 2009). Of more than 400 studies that the investigators screened, only 127 interventions could be evaluated for their efficacy according to the inclusion criteria specified by the researchers. Of these 127 studies, 41 showed evidence of a positive effect—that is, they could be classified as “most promising” (*n* = 12) or having “mixed or emerging” evidence (*n* = 29). A list of the school-based interventions identified as most promising is provided in the table.

Two-thirds of the most-promising interventions that were identified by Spoth and colleagues (2008, 2009) either were exclusively school based (*n* = 2) or included a large school-based component within a multiple-component or multiple-domain intervention (*n* = 6). Most-promising interventions were identified for all three age-groups studied. At the elementary-school level, interventions classified as most promising included the following:

- Seattle Social Development Project (Hawkins et al. 1991, 1992);
- Linking the Interests of Families and Teachers (Eddy et al. 2000, 2003);
- Raising Healthy Children (Brown et al. 2005; Catalano et al. 2003); and
- Preventive Treatment Program (Tremblay et al. 1996).

At the middle-school level, the most promising interventions included the following:

- Project Northland (Perry et al. 1996, 2002);
- Project STAR, or Midwestern Prevention Project (Chou et al. 1998; Pentz et al. 1989, 1990); and
- *keepin’ it REAL* (Hecht et al. 2003).

At the high-school level, only the Project Toward No Drug Abuse (Sussman et al. 2002) was classified as most promising, although Project Northland also has been implemented and shown to be successful with high-school students (Perry et al. 2002).

Other school-based programs that may be familiar to readers who conduct research in this area, such as Promoting Alternative Thinking Strategies (Kam et al. 2004; Riggs et al. 2006), Life Skills Training (Botvin et al. 1995; Spoth et al. 2005), and Project Alert (Ellickson and Bell 1990; Ellickson et al. 2003) were identified as either having mixed (e.g., Life Skills Training, Project Alert) or emerging (e.g., Promoting Alternative Thinking Strategies) evidence, along with 26 other interventions (Spoth et al. 2008, 2009). Seventeen of 29 “mixed or emerging evidence” interventions either were exclusively school based (*n* = 11) or included a school-based component (*n* = 6). (See the review by Spoth and colleagues [2008, 2009], as well as the original literature cited above for a more detailed description of these interventions.)

Although the review by Spoth and colleagues (2008, 2009) offers concrete examples of evidence-based interventions, it does not address why some school-based interventions were effective and others were not. Other recent literature reviews (Cuijpers 2002; Komro and Toomey 2002) and meta-analyses (e.g., Roona et al. 2003; Tobler et al. 2000) have examined this issue. The findings suggest that the following elements are essential to developing and implementing effective school-based alcohol prevention interventions:

- The interventions are theory driven, with a particular focus on the social-influences model, which emphasizes helping students identify and resist social influences (e.g., by peers and media) to use alcohol.
- The interventions address social norms around alcohol use, reinforcing that alcohol use is not common or acceptable among youth.
- The interventions build personal and social skills that help students resist pressure to use alcohol.
- The interventions use interactive teaching techniques (e.g., small-group activities and role plays) to engage students.
- The interventions use same-aged students (i.e., peer leaders) to facilitate delivery of the program.
- The interventions integrate additional components to connect other segments of the community (e.g., parents) to the program.
- The interventions are conducted across multiple sessions and multiple years to ensure that an adequate “dose” of prevention is received by students and schools.
- The interventions provide adequate training and support for program facilitators (i.e., teachers, students).
- The interventions are both culturally and developmentally appropriate for the students they serve.

Two projects that are examples of programs meeting the criteria noted above are Project Northland (Perry et al. 1996, 2002) and Communities that Care (Hawkins et al. 2009). These community-wide programs used evidence-based school curricula, supplemented with parental involvement, peer leadership, and community action to achieve reductions in the onset of alcohol use in early adolescence. Communities that Care is described in more detail in the article by Fagan and colleagues (pp. 167–174, in this issue) that focuses on community-based preventive interventions.

## Future Directions for School-Based Alcohol Prevention Interventions

Although the understanding of effective interventions to prevent underage alcohol use has grown substantially over the last few decades, especially for school-based approaches, additional research is warranted to fill remaining gaps in the knowledge base. For example, the existing literature does not include sufficient evidence to support or refute the short- or long-term efficacy of school-based interventions in elementary-or high-school settings and does not fully address interventions for special populations, including culturally specific programming. These points are considered in more detail below as suggestions for future directions for school-based research. Readers are directed to the reviews by Spoth and colleagues (2008, 2009) for additional discussion of needed improvements in conducting and reporting this research.

### School-Based Interventions for Elementary-School and High-School Settings

As noted above, the majority of school-based alcohol prevention interventions have been conducted in middle schools. By comparison, far fewer interventions have been developed for elementary schools and high schools. In the review by Spoth and colleagues (2008), only one school-based intervention for high-school students could be classified as most promising, and only one could be classified as having mixed or emerging evidence. However, alcohol use is particularly problematic during the high-school years. Nationwide, almost half of high-school seniors report consuming alcohol in the previous month, and one-third were drunk in the last month (Johnston et al. 2010). Accordingly, sustained intervention throughout high school likely is necessary to maintain any changes in developmental trajectories of alcohol use achieved through interventions delivered in middle school, as was demonstrated by the high-school component of Project Northland (Perry et al. 2002). Further efforts to curb more problematic patterns of alcohol use, such as binge drinking, also are warranted during this period (Spoth et al. 2008).

Additional efforts to design, develop, and test school-based interventions for younger age-groups (e.g., “tweens”) are needed as well, given that school-based interventions seem to be most efficacious when delivered as a primary prevention program, with the strongest effects found in youth who have not yet begun to experiment with alcohol (Perry et al. 1996). Early onset of alcohol use during the teen or pre-teen years is of great concern because it can have substantial physical, social, and emotional health consequences for children and adolescents (e.g., Ellickson et al. 2003; Grant and Dawson 1997), including impairment of key brain functions and development (Squeglia et al. 2009). Of note, a large proportion of young adolescents use or begin to use alcohol before middle school. For example, in Project Northland Chicago, 17 percent of these urban sixth graders had started drinking alcohol before they entered middle school (Pasch et al. 2009), and the proportion was even higher (i.e., 37 percent) in rural Minnesota, in the original Project Northland; moreover, these students were much less responsive to the intervention than students who had not begun drinking (Perry et al. 1996). These high rates of early alcohol use make it worthwhile to introduce earlier, universal approaches to alcohol prevention. For example, Spoth and colleagues (2008) suggested intervening in grades 3, 4, and 5; however, none of the existing school-based programs aimed at the later elementary-school years met the criteria for inclusion in their review.

## School-Based Interventions for Special Populations

To date, the large majority of school-based interventions have been implemented with primarily White urban and suburban youth. The problem of alcohol use, however, is not limited to these populations. Alcohol use rates among school-going youth often are higher in rural settings, especially rates of binge drinking (i.e., five or more drinks in one sitting in the last 2 weeks) and drunkenness (Johnston et al. 2010). With respect to ethnic groups, rates of alcohol use among Hispanic eighth graders exceed those of White eighth graders, followed by African Americans (Johnston et al. 2010). Accordingly, the need for alcohol use prevention interventions tailored for these special populations is great. Although the body of research on this topic is growing, it requires even more attention. As Schinke and colleagues (2000) noted in a Cochrane review, culturally focused interventions may be an especially valuable approach to intervention over the long term. However, additional development and rigorous evaluation of this approach is required (Foxcroft et al. 2002).

In their review, Spoth and colleagues (2008) identified a few school-based alcohol prevention interventions specifically designed for special populations (e.g., minority youth, rural youth) with promising or emerging evidence. For example, *keepin’ it REAL* is a culturally grounded alcohol prevention program developed for and tested in Mexican and Mexican-American middle-school students (Hecht et al. 2003; Kulis et al. 2005). Instead of “translating” an existing school-based program originally designed for majority youth for use in this population, Hecht and colleagues (2003) crafted a successful program grounded from the beginning in ethnic norms and values. Their multicultural version, based on Latino, European-American, and African-American norms and values, was especially effective at reducing alcohol use over time (Kulis et al. 2005). Approaches like these that influence the deeper structure of an intervention might be necessary to effectively meet the needs of special populations as additional efforts are considered and subsequently undertaken to adapt existing evidence-based interventions for use in nonmajority, understudied groups.

Efforts to date to translate or adapt existing evidence-based interventions for special populations and settings have produced mixed results (Spoth et al. 2008). For example, the adaptation of Project Northland for use with a multiethnic population in Chicago was unsuccessful at changing alcohol use behaviors among those urban middle-school youth (Komro et al. 2008), even though the adaptation included not only surface-structure changes (e.g., changes in text and graphics) but also the deep-structure changes (e.g., incorporating culturally specific values and norms) alluded to above (Komro et al. 2004; Resnicow et al. 1999). The original Project Northland in Minnesota had pursued a more proximal approach to intervention, with staff who were housed at the schools and with special emphasis given to school- and after-school–based activities, supplemented with parental involvement (Perry et al. 1996). The Chicago adaptation, in contrast, placed more emphasis on more distal intervention strategies, using staff who were housed in the community and emphasizing community organization to reduce access to alcohol (Komro et al. 2008). The results achieved with the two variants of the intervention suggest that in middle-school school students may require a more focused, hands-on approach to alcohol prevention. On the other hand, the Chicago implementation may have been less successful because alcohol use was less of a concern or priority in this population (Komro et al. 2008). Thus, in the Minnesota sample, alcohol use was the most serious problem found in the region of the State where the intervention was implemented (Perry et al. 1996), whereas in the Chicago sample other concerns (e.g., regarding other drugs or violence) were more prominent. Therefore, community needs, priorities, and readiness—as well as the question of how these can be shaped successfully—need to be considered carefully as translation research unfolds.

A final program worthy of note is Drug Abuse Resistance Education (D.A.R.E.). Although reviews of this program consistently show that it has little if any impact on alcohol and drug use (Ennett et al. 1994), it continues to be widely used across the United States. To capitalize on the powerful dissemination mechanism of the D.A.R.E. program, Perry and colleagues (2003) developed and evaluated D.A.R.E. Plus, which was successful in reducing tobacco and alcohol use among boys. These positive outcomes were attributed to the “Plus” components, such as peer leadership, parental education, and neighborhood involvement, because the D.A.R.E. program alone did not demonstrate these outcomes (Perry et al. 2003).

## Conclusion

Alcohol remains the drug of choice among America’s adolescents, with rates of current (i.e., past 30-day) use that are more than double those of cigarette smoking and rates of annual use that far exceed the use of marijuana and other illicit drugs (Johnston et al. 2010). Because alcohol use is more prevalent, and thus more normative, it remains more resistant to change than these other types of drug use. As a consequence, reducing underage alcohol use will require sustained intervention across adolescence, with added attention given to special populations for which effective interventions are not yet available. School-based interventions can be an effective approach to prevention, at least in the short term (Komro and Toomey 2002; Spoth et al. 2008, 2009). But because alcohol use currently is so normative among both adolescents and adults in the United States, comprehensive interventions that address multiple domains of a young person’s social environment—including the family, school, and community—likely will be required to substantially alleviate this problem in the long term. Given the predominance of school in the lives of youth, using schools as a central coordinating institution for primary prevention and linking them to families, worksites, media, and community policies is an efficient public health approach to alcohol use prevention that also can be efficacious.

## Figures and Tables

**Table:** The Most Promising School-Based Alcohol Prevention Interventions Identified by Spoth and Colleagues (2008, 2009)

**Children younger than 10 years of age**
Linking the Interests of Families and Teachers
Raising Healthy Children
Seattle Social Development Project
**Adolescents ages 10 to 15 years**
*keepin*’ it REAL
Midwestern Prevention Project/Project STAR
Project Northland
**Older participants ages 16 to more than 20 years**
Project Toward No Drug Abuse
